# The Efficacy of Heat-Clearing (Qingre) and Detoxifying (Jiedu) Traditional Chinese Medicine Gargle for Chemotherapy-Induced Oral Mucositis: A Systematic Review and Meta-Analysis

**DOI:** 10.3389/fphar.2021.627628

**Published:** 2021-03-29

**Authors:** Zhixian Lin, Jiangfeng Chen, Sunya Han

**Affiliations:** ^1^ Department of Oncology, The Third People's Hospital of Hangzhou, Hangzhou, China; ^2^ Department of Integrated Traditional Chinese and Western Medicine Oncology Ward 1, Hangzhou Cancer Hospital, Hangzhou, China; ^3^ The First Clinical Medical College of Zhejiang Chinese Medical University, Hangzhou, China

**Keywords:** chemotherapy, oral mucositis, heat-clearing (qingre) and detoxifying (jiedu), traditional Chinese medicine, gargle, meta-analysis

## Abstract

**Objective:** Chemotherapy-induced oral mucositis (CIOM) is an extremely serious complication of cancer. In China, the heat-clearing (Qingre) and detoxifying (Jiedu) traditional Chinese medicine QRJD-TCM gargle has been widely used to treat CIOM. To date, no systematic evaluation has been conducted on the clinical efficacy of QRJD-TCM gargle in treating CIOM. The objective of this systematic review and meta-analysis was to evaluate the efficacy of QRJD-TCM gargle in the treatment of CIOM.

**Methods:** Relevant randomized controlled trials (RCTs) comparing QRJD-TCM gargle with conventional Western medicine mouthwash (CWMM) for CIOM were confirmed by systematically searching PubMed, Embase, Cochrane Library, China National Knowledge Infrastructure, Chinese Scientific Journal Database, Wanfang Database, and Sinomed until October 20, 2020. Two researchers independently assessed the risk of bias according to the Cochrane risk-of-bias criteria. Excel 2010 was used in setting up a database of extracted information, and RevMan 5.3.0 was used in analyzing included trial data. The composition of the QRJD-TCM gargle was evaluated.

**Results:** A total of 25 articles were included in this meta-analysis. Results showed that compared with CWMM, QRJD-TCM gargle can reduce the incidence of CIOM (OR = 0.23, 95% CI [0.18, 0.29], *p* < 0.00001) and severity of CIOM (grade I–II: OR = 0.36, 95%CI [0.28, 0.46], *p* < 0.00001; grade III–IV: OR = 0.15, 95%CI [0.09, 0.28], *p* < 0.00001). In addition, QRJD-TCM gargle improved the effective rate of CIOM (OR = 15.91, 95% CI [7.93, 31.89], *p* < 0.00001).

**Conclusion:** QRJD-TCM gargle is effective in preventing and treating CIOM. However, more standard, double-blind, and multicenter randomized controlled studies are needed to further confirm the efficacy of QRJD-TCM gargle in the prevention and treatment of CIOM.

## Introduction

Oral mucositis (OM) is one of the most common adverse reactions caused by chemotherapy; 30–40% of patients receiving normal chemotherapy regimens (excluding high-dose chemotherapy or combined radiotherapy) can develop OM (Lalla et al., 2014; Mahendran et al., 2018). OM is characterized by inflammation, edema, and erythema with or without ulcers (Chaveli-López, 2014; Chaveli-López and Bagán-Sebastián, 2016). Severe OM can lead to difficulty in eating, thereby affecting a patient's nutrition and quality of life. Thus, reducing the doses of chemotherapy drugs or stopping/postponing chemotherapy may be necessary. These measures ultimately affect the prognoses of cancer patients (Elting et al., 2003; Sonis, 2004).

Owing to the high incidence of chemotherapy-induced oral mucositis (CIOM) in cancer patients and its serious consequences, focusing on the treatment of CIOM is necessary. In clinical practice, CIOM has no standard treatment method, and symptomatic treatment is mainly used. The main methods of prevention and treatment include basic oral care, mouthwash, laser treatment, and cryotherapy (Lalla et al., 2014). Western medicine mouthwashes, which are commonly used in clinical practice and include normal saline, sodium bicarbonate, and chlorhexidine, generally have unstable drug properties and easily cause the imbalance of oropharyngeal flora (McGuire et al., 2013). Laser therapy has the advantages of promoting analgesia and ulcer surface healing, but the aspects of wavelength, power, and duration of treatment remain controversial (Ottaviani et al., 2013; Rezk-Allah et al., 2019). Cryotherapy inhibits the entry of chemotherapy drugs into mucosal tissues and thereby reduces the incidence of CIOM, but it potentially reduces the effectiveness of primary disease treatment; moreover, cold conditions causes psychological resistance (Askarifar et al., 2016; Daugėlaitė et al., 2019). Given the limitations of current treatments, supplementary and/or alternative drugs that alleviate CIOM symptoms are urgently needed.

Chinese herbal medicine has been widely used to treat CIOM (Gesa et al., 2013). According to the theory of traditional Chinese medicine (TCM), chemotherapy drugs belong to the “evil poison” and “drug poison” from the environment. The clinical manifestations of CIOM, including ulcers, redness, swelling, and pain, are the characteristics of TCM heat toxin syndrome. The classic TCM treatment for heat toxin syndrome is heat clearing and detoxifying (Jin et al., 2009; Pan, 2012). In the heat clearing and detoxifying method, a combination of Chinese herbal medicines with clearing heat and/or detoxifying effects is used as the principal drug. Zhou et al. showed that Jiedu Yuyang decoction with gargle is effective in preventing and treating CIOM, exerts a significant effect on wound healing rate, and promotes swelling and pain relief (Zhou et al., 2019). Another study demonstrated the efficacy and safety of heat clearing (Qingre) and detoxifying (Jiedu) traditional Chinese medicine (QRJD-TCM) gargle in the prevention and treatment of CIOM (Kong, 2020). QRJD-TCM gargle has the advantages of easy availability, low price, and causing few adverse reactions, and does not promote drug resistance. It can not only prevent the occurrence of CIOM but can also effectively control the development of CIOM (Li et al., 2012). However, no systematic review has reported the role of QRJD-TCM gargle in reducing CIOM. To confirm the clinical efficacy of QRJD-TCM gargle in CIOM treatment and provide a reference for clinical practice, we systematically collected studies on QRJD-TCM treatment for CIOM and conducted a systematic review and meta-analysis.

## Materials and Methods

We conducted this meta-analysis according to the Preferred Reporting Items for the Systematic Review and Meta-Analysis (PRISMA) guidelines. The data were obtained from published trials.

### Search Strategy

PubMed, Embase, Cochrane Library, China National Knowledge Infrastructure (CNKI), Chinese Scientific Journal Database (VIP), Wanfang Database, and Sinomed were systematically searched until October 20, 2020. The following search terms were used: “traditional Chinese medicine,” “Chinese herbal medicine,” “herbs,” “heat-clearing,” “Qingre,” “detoxifying,” “Jiedu,” “chemotherapy,” “oral mucositis,” and “oral ulcer.” Details of the search strategies are available in Supplementary Materials 1. In addition, we reviewed references to eligible studies and related systematic reviews to search relevant articles that may have been missed in the online searches.

### Inclusion Criteria

Studies that met all of the following criteria were included:

1. Study design was randomized controlled trial (RCT).
2. Patients were diagnosed with CIOM according to the China Cancer Symptom Management Practice Guide for Oral Mucositis (Ma et al., 2020), and limitations related to age, ethnicity, gender, cancer type, chemotherapy regimen, or chemotherapy drug dose were nonexistent.
3. Patients in the trial group used various kinds of QRJD-TCM gargle. Limitations regarding prescription composition, dosage, or course of treatment were nonexistent.
4. Patients in the control group received CWMM. Limitations regarding specific drugs, doses, concentrations, or courses of treatment were nonexistent.
5. The studies must include outcome indicators related to CIOM. 

### Exclusion Criteria

Studies that met any of the following criteria were excluded:

1. The full text cannot be retrieved electronically, manually, or by e-mail.
2. Nonrandomized controlled trials, such as meta-analysis, retrospective studies, case reports, trial studies, and conference abstracts.
3. The patients with OM received radiotherapy or combined chemotherapy.
4. The trial group was non-QRJD-TCM intervention.
5. The intervention method of the trial group did not adopt the use of a gargle.
6. Studies that lack outcome data or cannot be analyzed.

### Study Selection and Data Extraction

Three independent reviewers (ZXL, JFC, and SYH) searched and screened the literature according to the research protocol. In the case of discrepancies, the final decision would be made through consensus. We extracted information from the included studies, including general information (first author, country, and year of publication), characteristics of participants (age, gender, sample size, and course of treatment), details of the QRJD-TCM (name of the prescription and composition), and characteristics of the control group and main outcomes.

### Risk of Bias Assessment

Two researchers (ZXL and JFC) independently assessed the risk of bias for each trial with the tool of Cochrane Collaboration (Higgins et al., 2011). The bias risk assessment tool contained the following assessment tools: random sequence generation (selection bias), allocation concealment (selection bias), blinding of participants and personnel (performance bias), blinding of outcome assessment (detection bias), incomplete outcome data (attrition bias), selective reporting (reporting bias), and other bias. After the risk of bias was assessed, each study was classified as “low risk of bias,” “unclear risk of bias,” or “high risk of bias” (Higgins and Green, 2008). Any disagreement was settled through consultation with the third researcher.

### Data Analysis

The RevMan (Review Manager 5.3) statistical software provided by Cochrane Collaboration was used for data analysis (Cochrane, 2012). Counting data were presented as odds ratio (OR) with a 95% confidence interval (CI). Chi-square test and I-square (I^2^fn2) index were used in testing heterogeneity. When *p* ≤ 0.05 and I^2^ ≥ 50%, the random effect model was used. When *p* ≥ 0.05 and I^2^ ≤ 50%, the fixed effect model was used. The sources of heterogeneity were analyzed, and the factors that led to heterogeneity were analyzed through subgroup analysis. A *p* value of ≤0.05 was considered statistically significant, and all tests were two-sided tests. In addition, we used funnel plots to assess the existence of publication bias and performed Egger’s tests with STATA v16.0. Sensitivity analysis was performed by removing individual studies, and the stability of the results was assessed.

## Results

### Search Results

**FIGURE 1:**
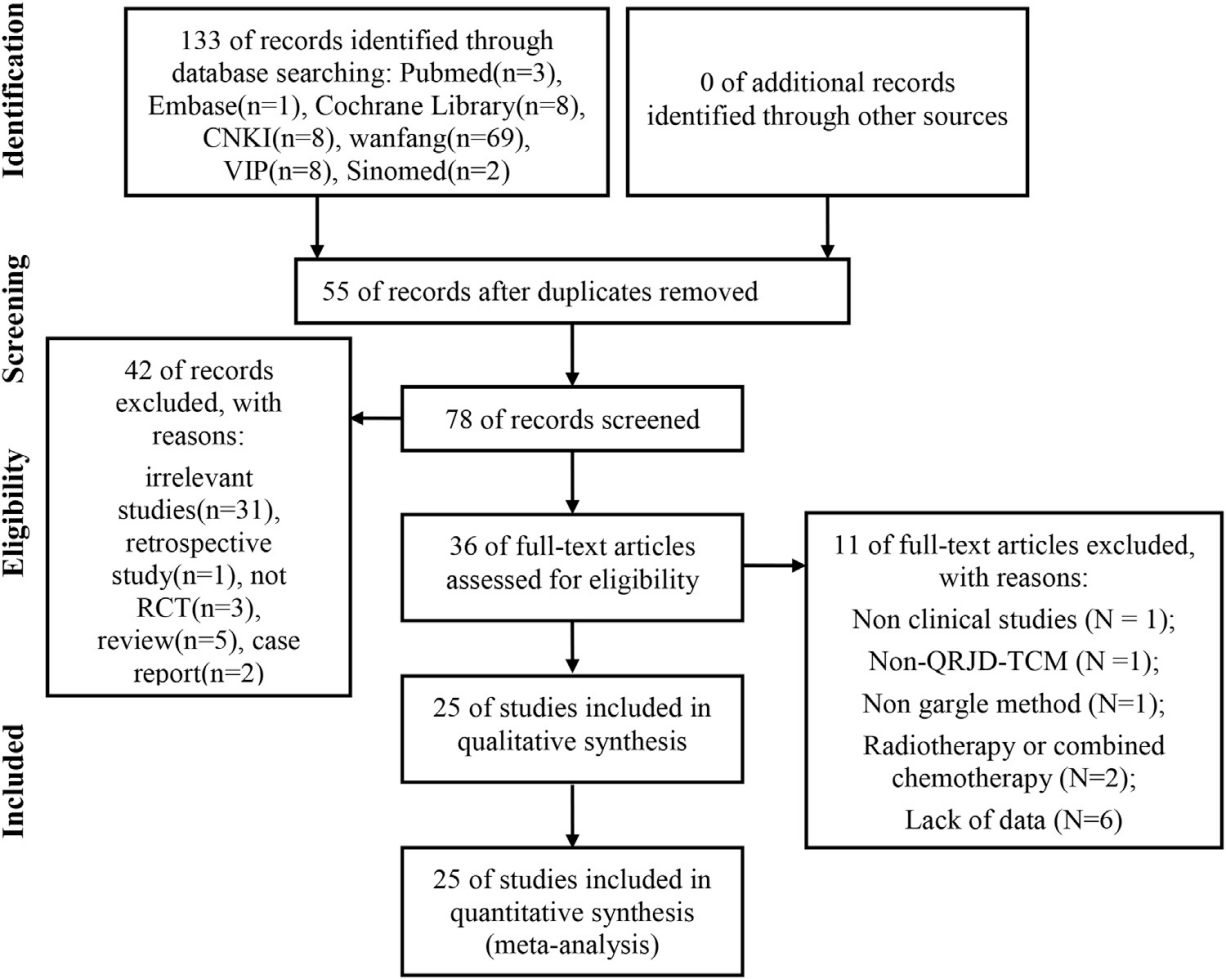
Flow chart of study identification and selection.

A total of 133 articles (the Cochrane Library [*n* = 8], PubMed [*n* = 3], embase [*n* = 1], Sinomed [*n* = 2], CNKI [*n* = 42], VIP [*n* = 8], and Wanfang Data [*n* = 69]) were retrieved. After we used the EndNote software to delete duplicate articles, we retained 78 studies for further confirmation. Then, by reading the titles and abstracts, 42 articles were excluded because of obvious ineligibility (31 irrelevant studies, one retrospective study, three nonrandomized controlled trials, five reviews, and two case reports). Finally, after reading the full text of the remaining 36 articles, we further removed 11 studies because of at least one of the following reasons: nonclinical studies (*n* = 1); non-QRJD-TCM (*n* = 1); intervention method of the trial group did not adopt the use of a gargle (*n* = 1); participants receiving radiotherapy or combined chemotherapy (*n* = 2); and lack of data (*n* = 6). Finally, we included 25 eligible studies for comprehensive analysis. The screening process is shown in Figure 1.

### Characteristics of Included Studies

Table 1 summarizes the basic characteristics of the 25 RCTs. One of the studies was conducted in Brazil (Reis et al., 2016), and another one was performed in Turkey (Ezgi et al., 2016). The other studies were carried out in China (23 trials). A total of 2,287 patients were recruited (1,151 in the trial group and 1,136 in the control group), with a sample size of 38 to 180 for a single trial. Patient age ranged from 5 to 83 years. The course of treatment lasted from 5 to 21 days. The control group received CWMM treatment, including normal saline solution (Hang, 2012; Lu and Zhang, 2014; Wang and Hang, 2014; Ezgi et al., 2016; Zhong, 2016), Xilei san (Pan and Yan, 2016; Yan et al., 2016) and cetylpyridinium chloride gargle (Wang et al., 2011; Kong, 2020). The trial group used QRJD-TCM gargle, including Yinhuang gargle (Hang, 2012; Wang and Hang, 2014; Zhong, 2016), and Daihua decoction (Pan and Yan, 2016; Yan et al., 2016). Nineteen studies reported the incidence of CIOM (Wang, 2002; Fang, 2012; Hang, 2012; Zhang, 2012; Duan et al., 2013; He et al., 2013; Xu, 2013; He, 2014; Lu and Zhang, 2014; Wang and Hang, 2014; He, 2015; Ezgi et al., 2016; Reis et al., 2016; Zhong, 2016; Zou et al., 2016; Li, 2018; Cheng, 2019; Zhou et al., 2019; Kong, 2020), 17 studies reported CIOM grade (Wang, 2002; Fang, 2012; Hang, 2012; Zhang, 2012; Duan et al., 2013; He et al., 2013; Xu, 2013; He, 2014; Wang and Hang, 2014; He, 2015; Ezgi et al., 2016; Reis et al., 2016; Zhong, 2016; Zou et al., 2016; Cheng, 2019; Zhou et al., 2019; Kong, 2020), and eight studies reported the effective rate of CIOM (Jin et al., 2009; Duan et al., 2013; Luo and Yang, 2013; Zhang, 2015; Pan and Yan, 2016; Yan et al., 2016; Wang et al., 2011; Kong, 2020).

**TABLE 1:** Baseline characteristics of included studies.

First author (year)	Country	Age/years	Sex		Sample size		Course of treatment/day	Intervention measure			Outcome
			Male	Female	Trial group	Control group		Trial group		Control group	
								Name of the prescription	Composition		
Zhang (2012)	China	26–77	69	57	60	66	15	Compound Scutellaria baicalensis Georgi gargle	Scutellaria baicalensis Georgi [Lamiaceae], Phellodendron chinense C.K.Schneid. [Rutaceae], Mentha *canadensis* L. [Lamiaceae], Paeonia × suffruticosa Andrews [Paeoniaceae], Corydalis yanhusuo (Y. H. Chou & Chun C.Hsu) W.T.Wang ex Z. Y. Su & C.Y.Wu [Papaveraceae], Carthamus tinctorius L. [Asteraceae], Prunus persica (L.) Batsch [Rosaceae]	Nystatin tablets	1. Incidence of oral mucositis 2. Mucositis severity
Zhou et al. (2019)	China	27–83	48	42	45	45	10	Jiedu Yuyang decoction	Lycium barbarum L. [Solanaceae], Lonicera japonica Thunb. [Caprifoliaceae], Chrysanthemum indicum L. [Asteraceae], Oroxylum indicum (L.) Kurz [Bignoniaceae], Lasiosphaera fenzlii Retch. [Lycoperdaceae], Asarum heterotropoides F.Schmidt [Aristolochiaceae]	Kangfuxin liquid	1. Incidence of oral mucositis 2. Mucositis severity
He (2015)	China	18–65	48	32	40	40	21	Jinyu gargle	*Lonicera japonica* Thunb. [Caprifoliaceae], Mentha *canadensis* L. [Lamiaceae], Polygonatum odoratum (Mill.) Druce [Asparagaceae], Glycyrrhiza inflata Batalin [Fabaceae]	Gentamicin and 4% sodium bicarbonate solution	1. Incidence of oral mucositis 2. Mucositis severity
Cheng (2019)	China	38–81	107	73	90	90	14	Shuanghuang yin	*Lonicera japonica* Thunb. [Caprifoliaceae], Taraxacum mongolicum Hand.-Mazz. [Asteraceae], Lophatherum gracile Brongn. [Poaceae], Blumea balsamifera (L.) DC. [Asteraceae], Mentha *canadensis* L. [Lamiaceae]	0.9% Sodium chloride solution, Vitamin B12 and Vitamin B2	1. Incidence of oral mucositis 2. Mucositis severity
Li (2018)	China	5–14	42	38	40	40	7	Wuji san	*Taraxacum* mongolicum Hand.-Mazz. [Asteraceae], Glycyrrhiza inflata Batalin [Fabaceae], Bletilla striata (Thunb.) Rchb.f. [Orchidaceae]	1% Sodium bicarbonate solution	1. Incidence of oral mucositis 2. Mucositis severity
Fang (2012)	China	36–76	38	52	45	45	NP	Solidago decurrens Lour. [Asteraceae] gargle	Solidago decurrens Lour. [Asteraceae]	Gentamicin and normal saline	1. Incidence of oral mucositis 2. Mucositis severity
Hang (2012)	China	45–65	68	52	40	40	8	Yinhuang gargle	*Lonicera japonica* Thunb. [Caprifoliaceae], Scutellaria baicalensis Georgi [Lamiaceae], Coptis chinensis Franch. [Ranunculaceae], Phellodendron chinense C.K.Schneid. [Rutaceae], Scrophularia ningpoensis Hemsl. [Scrophulariaceae], Prunus mume (Siebold) Siebold & Zucc. [Rosaceae], Glycyrrhiza inflata Batalin [Fabaceae], Vigna radiata (L.) R.Wilczek [Fabaceae]	Normal saline	1. Incidence of oral mucositis 2. Mucositis severity
Zhong (2016)	China	NP	60	40	52	48	8	Yinhuang gargle	*Lonicera japonica* Thunb. [Caprifoliaceae], Scutellaria baicalensis Georgi [Lamiaceae],Coptis chinensis Franch. [Ranunculaceae], Phellodendron chinense C. K. Schneid. [Rutaceae], Scrophularia ningpoensis Hemsl. [Scrophulariaceae], Prunus mume (Siebold) Siebold & Zucc. [Rosaceae], Glycyrrhiza inflata Batalin [Fabaceae], Vigna radiata (L.) R.Wilczek [Fabaceae]	Normal saline	1. Incidence of oral mucositis 2. Mucositis severity
Xu (2013)	China	39–73	35	31	36	30	10	Yinxuan decoction	*Lonicera japonica* Thunb. [Caprifoliaceae], Scrophularia ningpoensis Hemsl. [Scrophulariaceae], Chrysanthemum indicum L. [Asteraceae], Ophiopogon japonicus (Thunb.) Ker Gawl. [Asparagaceae]	Iodine volts and normal saline	1. Incidence of oral mucositis 2. Mucositis severity
He (2014)	China	43–68	37	39	38	38	NP	No	Scrophularia ningpoensis Hemsl. [Scrophulariaceae], Rehmannia glutinosa (Gaertn.) DC. [Orobanchaceae], Ophiopogon japonicus (Thunb.) Ker Gawl. [Asparagaceae], Lonicera japonica Thunb. [Caprifoliaceae], Isatis tinctoria L. [Brassicaceae] (Banlangen), Sophora tonkinensis Gagnep. [Fabaceae], Coptis chinensis Franch. [Ranunculaceae], Phellodendron chinense C.K.Schneid. [Rutaceae], Astragalus mongholicus Bunge [Fabaceae], Rhus chinensis Mill. [Anacardiaceae], Blumea balsamifera (L.) DC. [Asteraceae]	0.9% Sodium chloride solution and gentamycin	1. Incidence of oral mucositis 2. Mucositis severity
Lu and Zhang (2014)	China	39–57	22	38	30	30	10	No	*Lonicera japonica* Thunb. [Caprifoliaceae], Forsythia suspensa (Thunb.) Vahl [Oleaceae], Taraxacum mongolicum Hand.-Mazz. [Asteraceae], Solidago decurrens Lour. [Asteraceae], Chrysanthemum indicum L. [Asteraceae], Rosa multiflora Thunb. [Rosaceae]	Normal saline	1. Incidence of oral mucositis 2. Mucositis severity
Wang (2002)	China	31–68	62	85	76	71	NP	No	Phellodendron chinense C.K.Schneid. [Rutaceae], Rhus chinensis Mill. [Anacardiaceae], Verbena officinalis L. [Verbenaceae], Senegalia catechu (L.f.) P.J.H.Hurter & Mabb. [Fabaceae], Forsythia suspensa (Thunb.) Vahl [Oleaceae], Blumea balsamifera (L.) DC. [Asteraceae]	Dobell's solution	1. Incidence of oral mucositis 2. Mucositis severity
He et al. (2013)	China	31–86	34	26	30	30	14	No	*Taraxacum* mongolicum Hand.-Mazz. [Asteraceae], Polygonatum cyrtonema Hua [Asparagaceae], Portulaca oleracea L. [Portulacaceae], Dictamnus dasycarpus Turcz. [Rutaceae], Kochia scoparia (L.) Schrad. [Amaranthaceae], Lonicera japonica Thunb. [Caprifoliaceae]	Metronidazole and sodium chloride injection	1. Incidence of oral mucositis 2. Mucositis severity
Zou et al. (2016)	China	5–75	43	37	40	40	14	Compound Scutellaria baicalensis Georgi gargle	Scutellaria baicalensis Georgi [Lamiaceae], Syringa pinnatifolia Hemsl. [Oleaceae], Mentha *canadensis* L. [Lamiaceae], Panax quinquefolius L. [Araliaceae], Magnolia officinalis Rehder & E.H.Wilson [Magnoliaceae], Prunus persica (L.) Batsch [Rosaceae], Phellodendron chinense C.K.Schneid. [Rutaceae]., Corydalis yanhusuo (Y.H.Chou & Chun C.Hsu) W.T.Wang ex Z.Y.Su & C.Y.Wu [Papaveraceae], Paeonia × suffruticosa Andrews [Paeoniaceae]	0.9% normal saline and gentamicin	1. Incidence of oral mucositis 2. Mucositis severity
Wang and Hang (2014)	China	39–71	56	44	50	50	14	Yinhuang gargle	*Lonicera japonica* Thunb. [Caprifoliaceae], Coptis chinensis Franch. [Ranunculaceae], Scutellaria baicalensis Georgi [Lamiaceae], Phellodendron chinense C.K.Schneid. [Rutaceae], Prunus mume (Siebold) Siebold & Zucc. [Rosaceae], Scrophularia ningpoensis Hemsl. [Scrophulariaceae], Glycyrrhiza inflata Batalin [Fabaceae], Vigna radiata (L.) R.Wilczek [Fabaceae]	Normal saline	1. Incidence of oral mucositis 2. Mucositis severity
Reis et al. (2016)	Brazil	42–79	20	18	20	18	15	Chamomile infusion	A cup of ice chips made with Matricaria chamomilla L. [Asteraceae] at 2.5%	A cup of ice chips made with pure water	1. Incidence of oral mucositis 2. Mucositis severity
Ezgi et al. (2016)	Turkey	18–65	24	36	30	30	5	Sage tea–thyme–peppermint hydrosol oral rinse	Salvia officinalis L. [Lamiaceae], Thymus mongolicus (Ronniger) Ronniger [Lamiaceae], Mentha *canadensis* L. [Lamiaceae]	Normal saline	1. Incidence of oral mucositis 2. Mucositis severity
Duan et al. (2013)	China	6–61	69	46	58	57	7	Compound Yinju decoction	Compound Yinju Mixture (produced by the preparation Room of Qingyang Hospital of Traditional Chinese Medicine, NO 20090112)	Chlorhexidine gargle	1. Incidence of oral mucositis 2. Mucositis severity 3. Effective rate
Kong (2020)	China	21–57	38	22	31	31	10	No	Chrysanthemum indicum L. [Asteraceae], Lonicera japonica Thunb. [Caprifoliaceae], Viola philippica Cav. [Violaceae], Bletilla striata (Thunb.) Rchb.f. [Orchidaceae], Mentha *canadensis* L. [Lamiaceae], Glycyrrhiza inflata Batalin [Fabaceae]	Cetylpyridinium chloride gargle	1. Incidence of oral mucositis 2. Mucositis severity 3. Effective rate
Pan and Yan (2016)	China	20–65	73	47	60	60	10	Daihua decoction	Isatis tinctoria L. [Brassicaceae] (Qingdai), Lonicera japonica Thunb. [Caprifoliaceae], Taraxacum mongolicum Hand.-Mazz. [Asteraceae], Angelica dahurica (Hoffm.) Benth. & Hook.f. ex Franch. & Sav. [Apiaceae], Bletilla striata (Thunb.) Rchb.f. [Orchidaceae], Panax notoginseng (Burkill) F.H.Chen [Araliaceae], Scutellaria baicalensis Georgi [Lamiaceae], Sepiae Endoconcha [Sepiidae]	Xilei san	Effective rate
Yan et al. (2016)	China	20–65	64	36	50	50	10	Daihua decoction	Isatis tinctoria L. [Brassicaceae] (Qingdai), Lonicera japonica Thunb. [Caprifoliaceae], Taraxacum mongolicum Hand.-Mazz. [Asteraceae], Angelica dahurica (Hoffm.) Benth. & Hook.f. ex Franch. & Sav. [Apiaceae], Bletilla striata (Thunb.) Rchb.f. [Orchidaceae], Panax notoginseng (Burkill) F.H.Chen [Araliaceae], Scutellaria baicalensis Georgi [Lamiaceae], Sepiae Endoconcha [Sepiidae]	Xilei san	Effective rate
Wang et al. (2011)	China	42–83	36	24	30	30	7	Compound Qiju decoction	Lycium barbarum L. [Solanaceae], Lonicera japonica Thunb. [Caprifoliaceae], Oroxylum indicum (L.) Kurz [Bignoniaceae], Chrysanthemum indicum L. [Asteraceae]	Cetylpyridinium chloride gargle	Effective rate
Zhang (2015)	China	56^a^	0	101	51	50	7	Qingshengan decoction	Isatis tinctoria L. [Brassicaceae] (Daqingye), Scrophularia ningpoensis Hemsl. [Scrophulariaceae], Glycyrrhiza inflata Batalin [Fabaceae]	0.02% Furacillin and 1–3% hydrogen peroxide solution	Effective rate
Jin et al. (2009)	China	13–83	33	27	30	28	7	Shuizhongcao decoction	Bubali Cornu [Bovidae], calamitas urinae hominis, Callicarpa macrophylla Vahl [Lamiaceae]	Leucovorin and gentamycin	Effective rate
Luo and Yang (2013)	China	57^a^	92	66	79	79	7	Yinjuhua gargle	*Lonicera japonica* Thunb. [Caprifoliaceae], Chrysanthemum indicum L. [Asteraceae]	0.02% Furacillin and 1–4% sodium bicarbonate solution	Effective rate

^a^ Mean age.

### Risk of Bias Assessment

**FIGURE 2:**
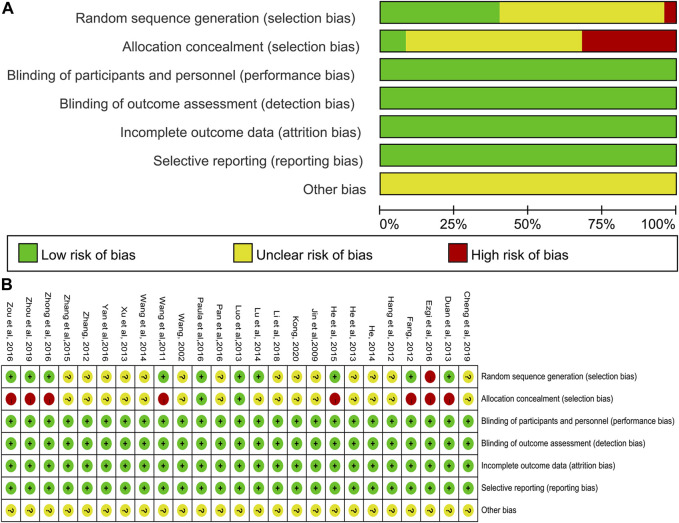
Risk of bias of included studies. **(A)** Risk of bias graph; **(B)** Risk of bias summary.

Among the 25 included studies, one study used computer-generated random numbers for random allocation (Luo and Yang, 2013), eight studies used random number tables (Wang et al., 2011; Fang, 2012; Duan et al., 2013; Lu and Zhang, 2014; He, 2015; Zhong, 2016; Zou et al., 2016; Zhou et al., 2019), and one study used the lottery method (Reis et al., 2016). The other studies claimed to have used randomization but have not reported details on how to randomize. Such studies were marked as “unclear risk.” Apart from two studies (Luo and Yang, 2013; Reis et al., 2016), these studies did not mention the allocation concealment method used. None of the included studies stated whether or not blinding was used but considered that these trials all used objective outcome indicators. Given that the results were not altered by researchers and participants, the studies were identified as “low-risk.” In addition, all the RCTs included had no incomplete data and selective reports, so both of them were marked as “low risk”. None of the studies had sufficient information that could be used in determining the presence of other significant risks of bias and were thus evaluated as “unclear risk.” In general, the overall quality of the methodology included in the study was moderate, and all the risk of bias assessment data are shown in Figure 2.

## Outcomes Measures

### Incidence of CIOM

**FIGURE 3:**
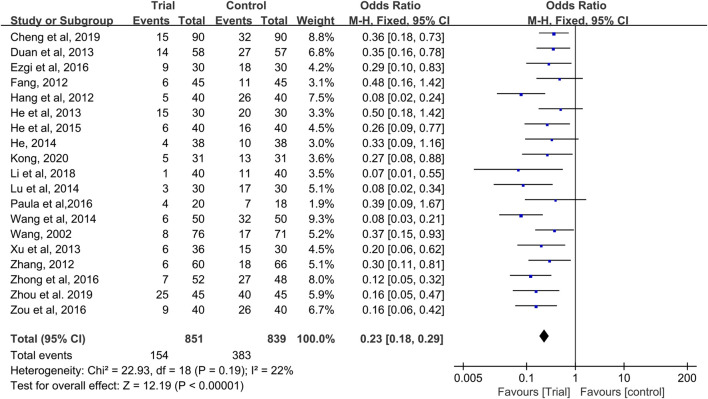
Forest plots of incidence of CIOM.

Nineteen studies reported CIOM data at any level during chemotherapy (Wang, 2002; Fang, 2012; Hang, 2012; Li et al., 2012; Zhang, 2012; Duan et al., 2013; He et al., 2013; Xu, 2013; He, 2014; Lu and Zhang, 2014; Wang and Hang, 2014; He, 2015; Ezgi et al., 2016; Reis et al., 2016; Zhong, 2016; Zou et al., 2016; Cheng, 2019; Zhou et al., 2019; Kong, 2020) with a total of 1,690 patients (851 cases in the QRJD-TCM therapy group and 839 cases in the CWMM treatment group). Given that data heterogeneity was not found (I^2^ = 22%, *p* = 0.19), the fixed effect model was used. This finding indicated that the QRJD-TCM gargle therapy group (154/851) was better than the CWMM treatment group (383/839) in reducing the incidence of CIOM (OR = 0.23, 95% CI [0.18, 0.29], *p* < 0.00001, Figure 3).

### Grade I–II CIOM

**FIGURE 4:**
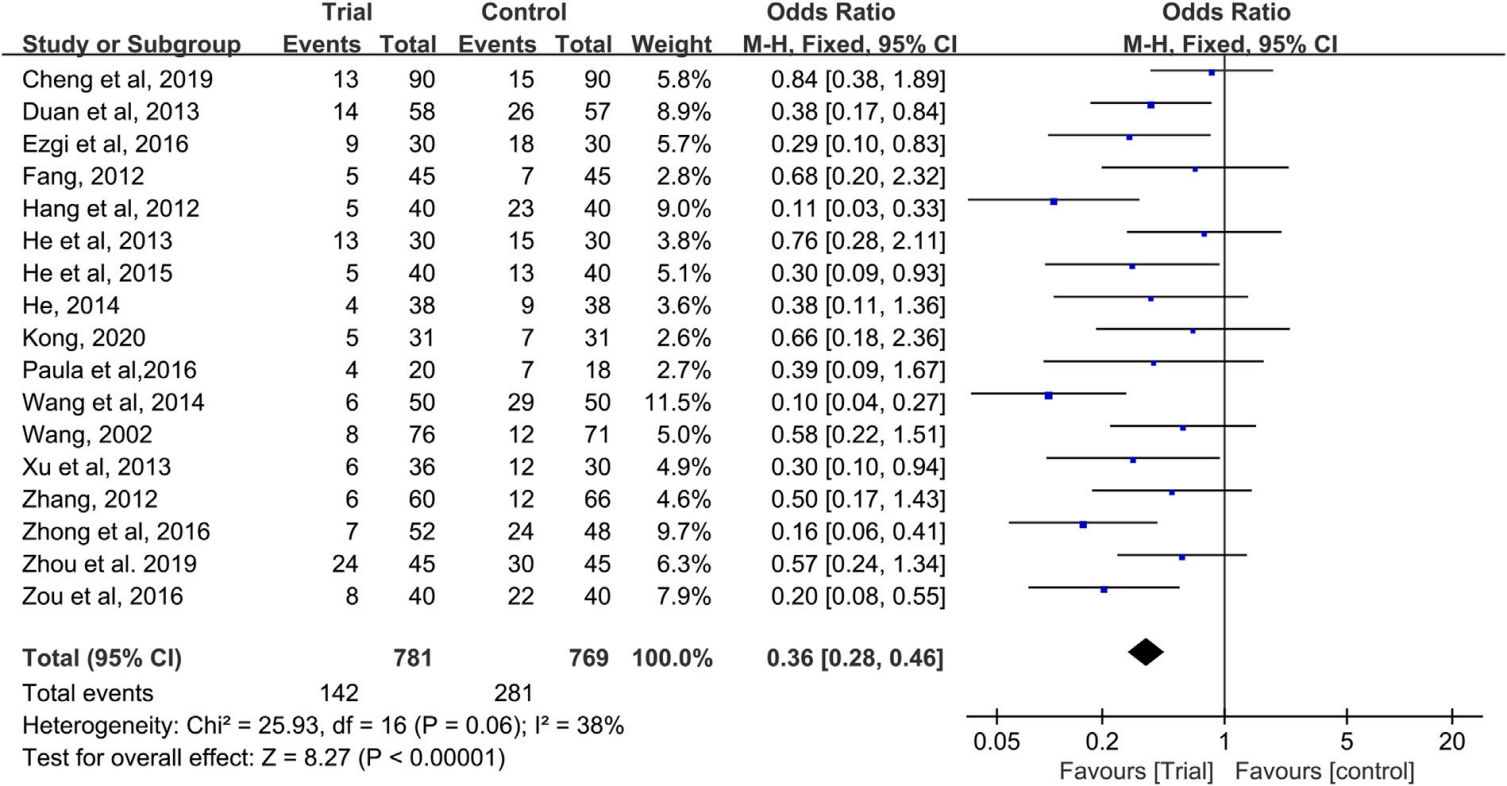
Forest plots of incidence of grade I-II CIOM.

Seventeen studies provided data on grade I–II CIOM (Wang, 2002; Fang, 2012; Hang, 2012; Zhang, 2012; Duan et al., 2013; He et al., 2013; Xu, 2013; He, 2014; Wang and Hang, 2014; He, 2015; Ezgi et al., 2016; Reis et al., 2016; Zhong, 2016; Zou et al., 2016; Cheng, 2019; Zhou et al., 2019; Kong, 2020), with a total of 1,550 patients (781 cases in the QRJD-TCM therapy group and 769 cases in the CWMM treatment group). No significant heterogeneity was found across the studies (I^2^ = 38%, *p* = 0.06) and thus the fixed effect model was used. The results showed that the incidence of grade I–II CIOM was significantly lower in the QRJD-TCM therapy group (142/781) than in the CWMM treatment group (281/769) (OR = 0.36, 95% CI [0.28, 0.46], *p* < 0.00001, Figure 4).

### Grade III–IV CIOM

**FIGURE 5:**
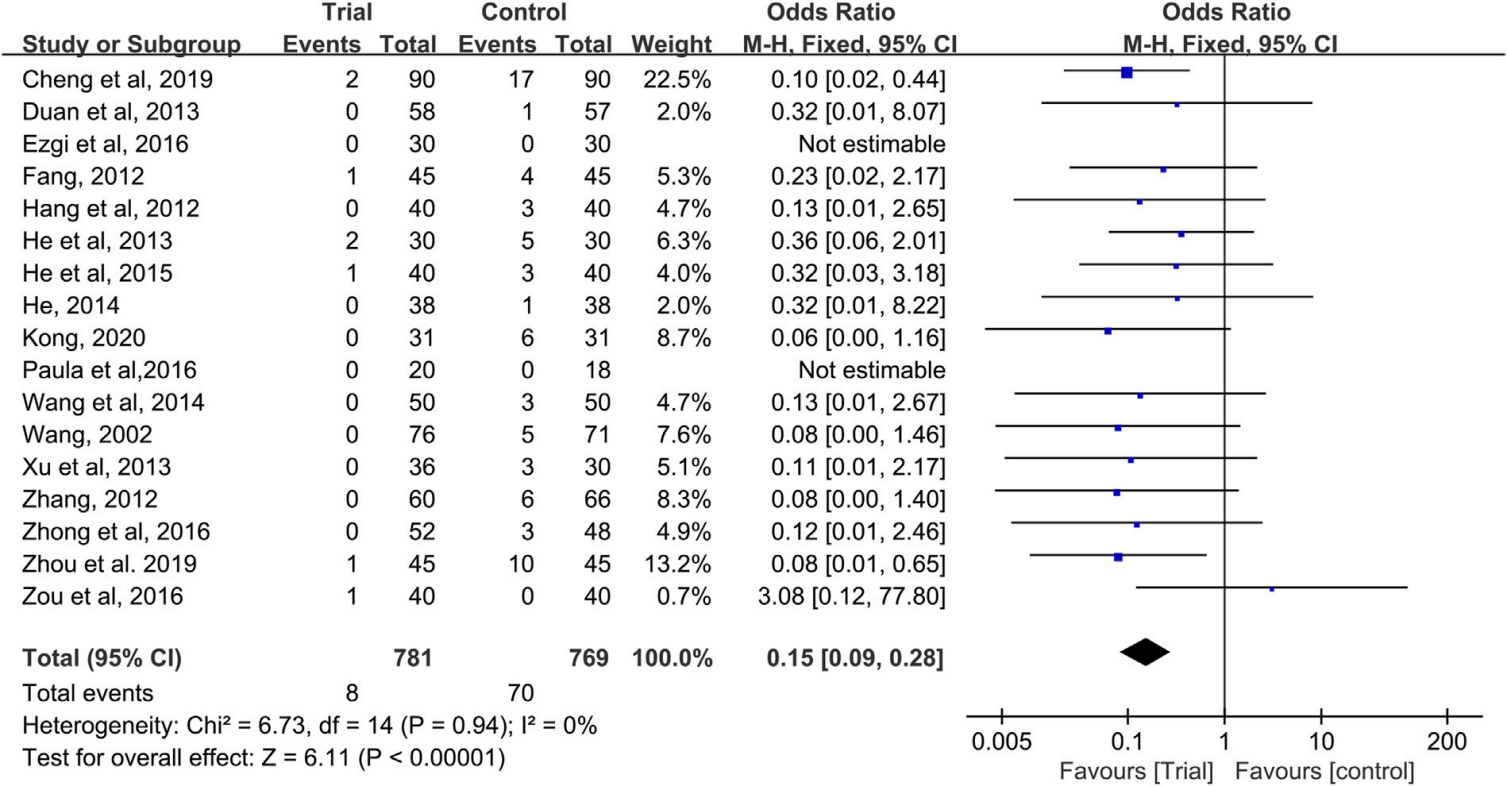
Forest plots of incidence of grade III-IV CIOM.

Seventeen studies provided data on grade III–IV CIOM (Wang, 2002; Fang, 2012; Hang, 2012; Zhang, 2012; Duan et al., 2013; He et al., 2013; Xu, 2013; He, 2014; Wang and Hang, 2014; He, 2015; Ezgi et al., 2016; Reis et al., 2016; Zhong, 2016; Zou et al., 2016; Cheng, 2019; Zhou et al., 2019; Kong, 2020), with a total of 1,550 patients (781 cases in the QRJD-TCM therapy group and 769 cases in the CWMM treatment group). No significant heterogeneity was found across the studies (I^2^ = 0%, *p* = 0.94) and thus the fixed effect model was used. The results showed that the incidence of grade III–IV CIOM was significantly lower in the QRJD-TCM therapy group (8/781) than in the CWMM treatment group (70/769) (OR = 0.15, 95% CI [0.09, 0.28], *p* < 0.00001, Figure 5).

### Effective Rate of CIOM

**FIGURE 6:**
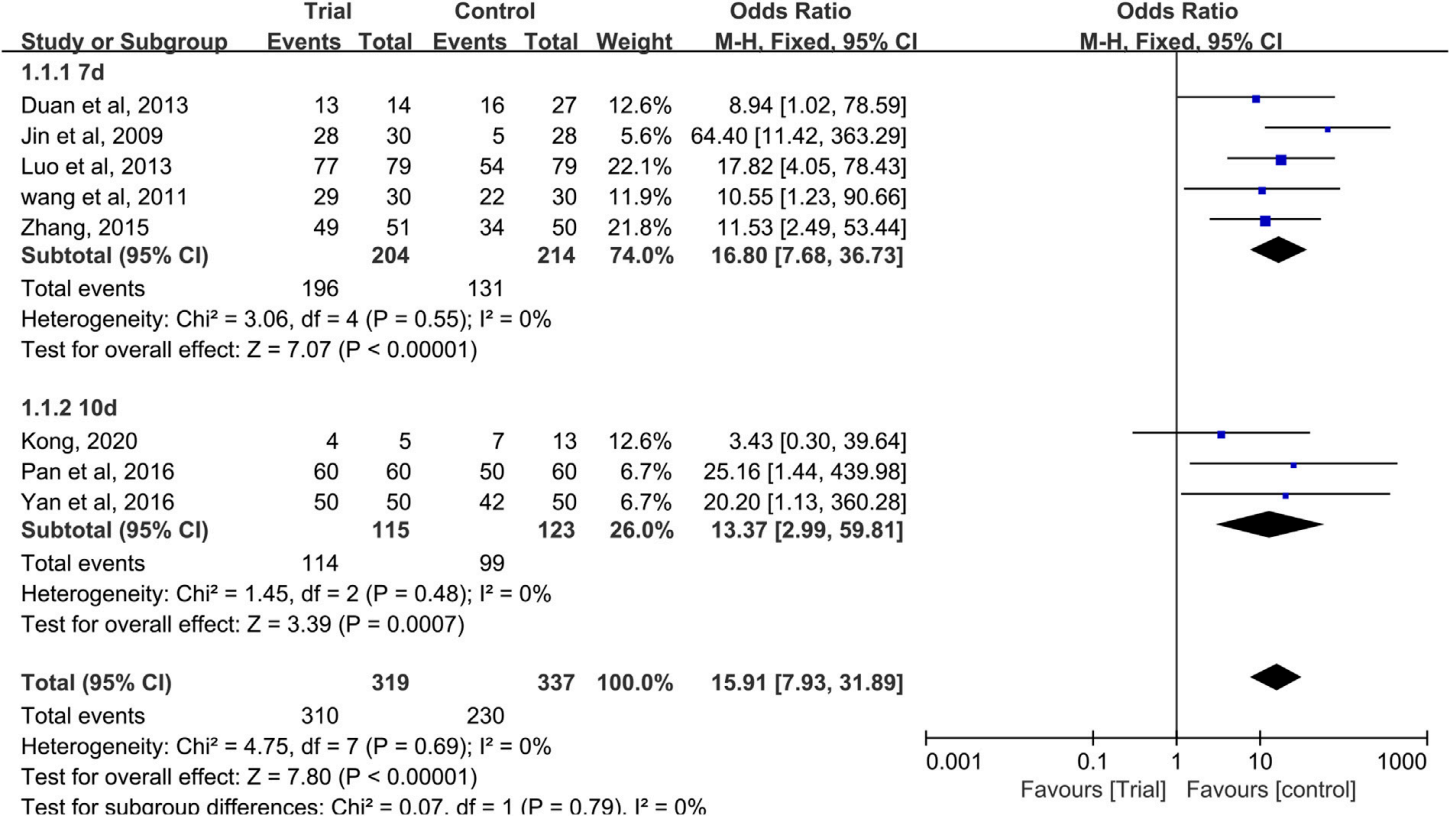
Forest plots of effective rate of CIOM including subgroup analysis based on the course of treatment.

Eight studies reported effective rates (Jin et al., 2009; Wang et al., 2011; Duan et al., 2013; Luo and Yang, 2013; Zhang, 2015; Pan and Yan, 2016; Yan et al., 2016; Kong, 2020), with a total of 656 patients (319 cases in the QRJD-TCM therapy group and 337 cases in the CWMM treatment group). Data heterogeneity was not found (I^2^ = 0%, *p* = 0.69), and thus the fixed effect model was applied. The results showed that the clinical effective rate was significantly higher in the QRJD-TCM gargle therapy group (310/319) than the CWMM treatment group (230/337; OR = 15.91, 95% CI [7.93, 31.89], *p* < 0.00001, Figure 6).

In these studies, effective rate subgroup analysis was performed according to different treatment times (7 or 10 days). Five trials (Jin et al., 2009; Wang et al., 2011; Duan et al., 2013; Luo and Yang, 2013; Zhang, 2015) compared the effective rates after 7 days of treatment, and three trials (Pan and Yan, 2016; Yan et al., 2016; Kong, 2020) compared the effective rates after 10 days of treatment. In the 7 and 10 days treatment duration subgroups, meta-analysis results indicated that QRJD-TCM gargle can improve the effective rates of CIOM (7 days: OR = 16.8, 95% CI [7.68, 36.73], *p* < 0.00001; 10 days: OR = 13.37, 95% CI [2.99, 59.81], *p* = 0.0007, Figure 6).

### Effect on Oral Pain

Four trials reported patients’ pain scores on a numeric rating scale. Among them, three trials (Wang et al., 2011; Zhang, 2015; Pan and Yan, 2016) reported patient's pain scores before and after treatment, and one trial (Zhou et al., 2019) reported the grade of pain relief after treatment. Therefore, we were unable to combine the results for meta-analysis. Overall, the pain relief rate of the trial group was better than that of the control group.

### High-Frequency Chinese Herbs

The composition of prescriptions of QRJD-TCM gargle included in the 25 studies was statistically analyzed. The Chinese herbs were ranked according to frequency of use, and high-frequency Chinese herbs were selected. The top four Chinese herbs were *Lonicera japonica* Thunb. [Caprifoliaceae] (17, 12.8%), Chrysanthemum indicum L. [Asteraceae] (8, 6%), Scutellaria baicalensis Georgi [Lamiaceae] (7, 5.3%), and Phellodendron chinense C.K.Schneid. [Rutaceae] (7, 5.3%), as shown in Table 2.

**TABLE 2:** High-frequency Chinese herbs.

English name		Chinese name		Counts		Frequency (%)	
*Lonicera japonica* Thunb. [Caprifoliaceae]		Jinyinhua		17		12.8	
Chrysanthemum indicum L. [Asteraceae]		Juhua		8		6	
Scutellaria baicalensis Georgi [Lamiaceae]		Huangqin		7		5.3	
Phellodendron chinense C.K.Schneid. [Rutaceae]		Huangbo		7		5.3	
*Taraxacum* mongolicum Hand.-Mazz. [Asteraceae]		Pugongying		6		4.5	
Mentha *canadensis* L. [Lamiaceae]		Bohe		6		4.5	
Scrophularia ningpoensis Hemsl. [Scrophulariaceae]		Xuanshen		6		4.5	
*Glycyrrhiza* inflata Batalin [Fabaceae]		Gancao		6		4.5	
Coptis chinensis Franch. [Ranunculaceae]		Huanglian		4		3	
Bletilla striata (Thunb.) Rchb.f. [Orchidaceae]		Baiji		4		3	

### Adverse Events

Among the 25 studies, one study (He, 2014) reported adverse events, including two cases of nausea and one case of gingival oozing in the trial group and one case of nausea and one case of gingival oozing in the control group, all of which were alleviated after symptomatic treatment. Two studies (Duan et al., 2013; Pan and Yan , 2016) clearly pointed out that no serious adverse events occurred during treatment. No adverse events were mentioned in the remaining studies.

### Publication Bias

**FIGURE 7:**
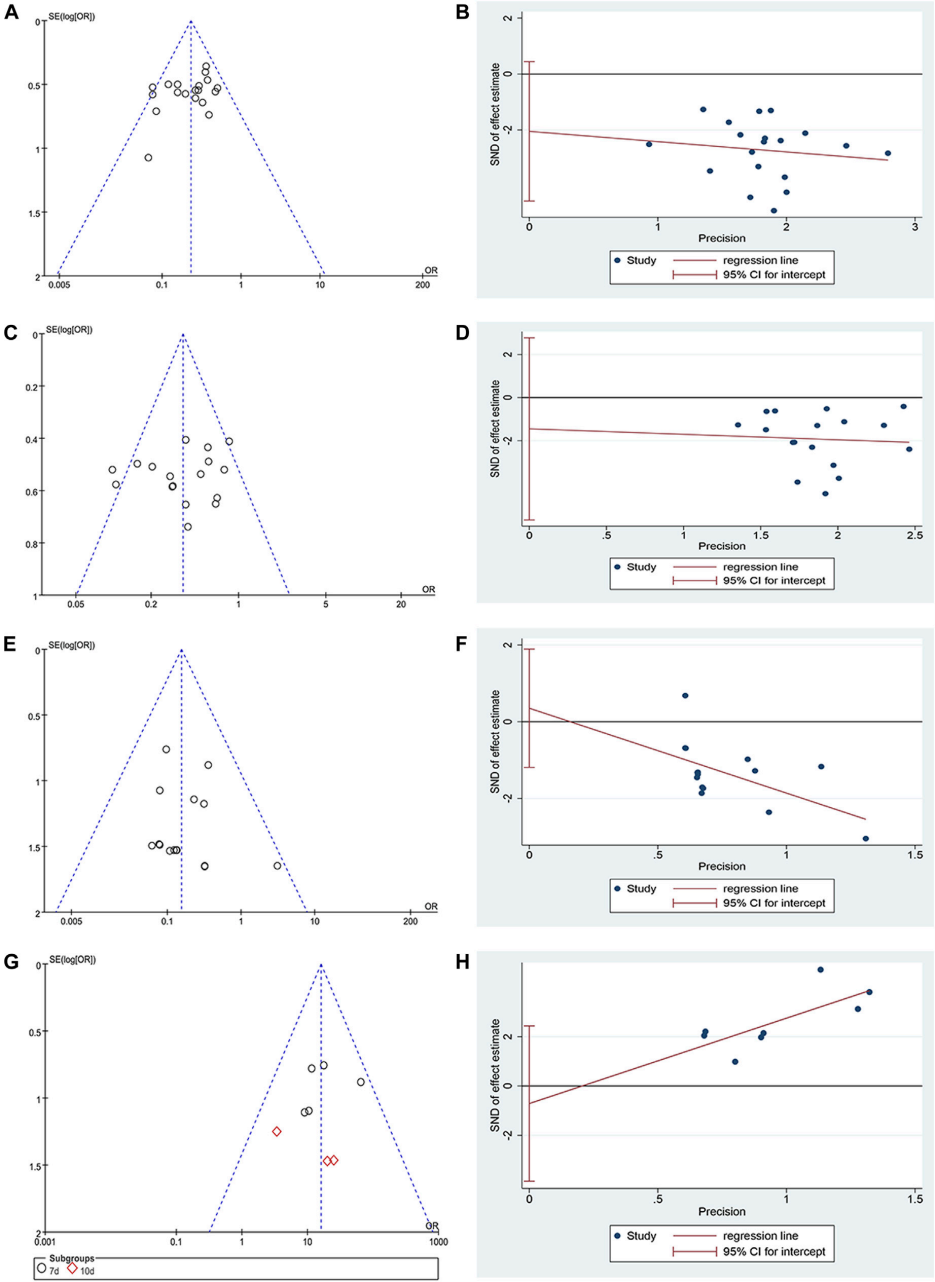
Publication bias plots. **(A)** Funnel plot of incidence of CIOM; **(B)** Egger’s plot of incidence of CIOM; **(C)** Funnel plot of grade I–II CIOM; **(D)** Egger’s plot of grade I–II CIOM; **(E)** Funnel plot of grade III–IV CIOM; **(F)** Egger’s plot of grade III–IV CIOM; **(G)** Funnel plot of effective rate of CIOM; **(H)** Egger’s plot of effective rate of CIOM.

A funnel plot and Egger’s test were used in analyzing publication bias. The funnel plot was drawn using the OR value of each result as the abscissa and SE (log[OR]) as the ordinate. Figures 7A,E,G show that the funnel shape was basically inverted and symmetrical. The result of Egger's test was incidence of CIOM: t = −1.73, *p* = 0.101; grade III–IV CIOM: t = 0.49, *p* = 0.629; effective rate of CIOM: t = −0.55, *p* = 0.6 (Figures 7B,F,H). The results of the two tests showed no obvious publication bias in the studies of incidence of CIOM, grade III–IV CIOM, and effective rate of CIOM. Figure 7C shows three studies (Hang, 2012; Wang and Hang, 2014; Cheng, 2019) at the edge of the funnel plot, indicating possible publication bias. The Egger's test result was grade I–II CIOM: *t* = −0.73, *p* = 0.476 (Figure 7D), indicating that the study of grade I–II CIOM had no obvious publication bias.

### Sensitivity Analysis

After we excluded each study, we found no significant change in the results, which all reached statistical significance. This finding indicated that the result data had good stability. After using different analytical models, we found that our conclusions were robust. These analysis results are provided in the supplementary materials (Supplementary Material 2).

## Discussion

As a disease caused by chemotherapy, CIOM can be alleviated or cured by using TCM gargle (Lockhart and Sonis, 1981). A far as we know, we are the first to conduct a systematic review and meta-analysis for studying the efficacy of QRJD-TCM gargle in preventing and treating CIOM. The analysis of 25 RCTs indicated that the prophylactic QRJD-TCM gargle can significantly reduce the incidence and the grade of CIOM, and therapeutic QRJD-TCM gargle can considerably increase the cure rate of CIOM. The results of the combined analysis of the incidence of CIOM showed heterogeneity among the studies possibly because of the age of the patients and the different chemotherapy regimens received. Studies have shown that the younger a patient is, the higher the chances that chemotherapy will cause adverse effects on oral health are (Cheng et al., 2011). The type, dosage, and frequency of chemotherapy drugs may have an impact on the occurrence and severity of OM, especially 5-fluorouracil, cyclophosphamide, cisplatin, and methotrexate; these chemotherapy drugs are involved in the emergence of changes in the oral mucosa (Ribeiro et al., 2019). The result of the combined analysis of CIOM grade showed that, although the included studies were the same, slight differences in heterogeneity between the two studies were found. Therefore, we speculated that this difference is related to the lack of consistency in the outcome indicator data of grade I–II CIOM. The heterogeneity of the research was less than 50%, indicating that the results of the study were still credible. In addition, the results of the combined analysis of the effective rate of CIOM showed that the clinical effective rate of the trial group was significantly higher than that of the control group. The subgroup analysis compared the cure rate after 7 and 10 days of treatment, and the results were consistent with the results above, suggesting that the course of treatment may have not affected the results of the study. Moreover, only one of the included studies reported adverse events, so QRJD-TCM gargle has been considered safe. Visual assessment of funnel plots and Egger’s test results showed no publication bias in any of the above studies. The consistency of these results enhanced the credibility of the conclusion that QRJD-TCM gargle can effectively prevent and treat CIOM. We believe that interventions using QRJD-TCM gargle can be used during the entire course of chemotherapy, that is, QRJD-TCM gargle can be used at the beginning of chemotherapy until the symptoms of OM disappears. This procedure not only can alleviate or even eliminate the symptoms of OM in patients but also ensures the smooth progress of chemotherapy.

According to the interference of chemotherapy on the division and maturation of epithelial cells, the pathogenesis of CIOM can be divided into direct and indirect mechanisms. The direct mechanism is the direct sensitivity of the oral mucosa to apoptosis induced by cytotoxic drug treatment (Lockhart and Sonis, 1981; Verdi, 1993). In the indirect mechanism, some proinflammatory factors that are indirectly toxic to the oral mucosa can promote the occurrence of CIOM, especially those produced by cytotoxic drugs, such as tumor necrosis factor-α (TNF-α), interleukin-1β (IL-1β), and interleukin-6 (IL-6) (Oronsky et al., 2018). Sonis (2010) proposed a five-stage model to describe the occurrence and development of CIOM: initiation, up-regulation and generation of messenger signals, signaling and amplification, ulceration, and healing.

Modern studies have proposed that the toxicity of heat clearing and detoxifying methods includes not only exogenous toxins, such as bacteria, viruses, and endotoxins, but also endogenous toxins, such as free radicals and inflammatory cytokines (Lu et al., 2004). Most QRJD-TCM have anti-inflammatory activity (Wang et al., 2018). In the included studies, the most commonly used QRJD-TCM are *Lonicera japonica* Thunb. [Caprifoliaceae], *Chrysanthemum indicum* L. [Asteraceae], *Scutellaria baicalensis* Georgi [Lamiaceae], and *Phellodendron chinense* C.K.Schneid. [Rutaceae]. *Lonicera japonica* Thunb. [Caprifoliaceae] has a broad-spectrum antibacterial effect (Zhao, 2007), and its extraction can inhibit LPS-induced NO secretion in RAW264.7 cells, down-regulate the release of IL-1β, IL-6, and TNF-α cytokines, and down-regulate the protein iNOS, COX2, and NF-κB p65 content to exert anti-inflammatory effects (Zeng et al., 2020). *Chrysanthemum indicum* L. [Asteraceae] extraction can inhibit the LPS-induced production of NO, PGE-2, TNF-α, and IL-1β and the expression of iNOS and COX-2 in macrophages (Cheon et al., 2009). *Scutellaria baicalensis* Georgi [Lamiaceae] extraction can effectively inhibit the inflammatory response of BV2 cells induced by LPS. Its anti-inflammatory mechanism is to inhibit the Toll-like receptor four inflammatory pathway activated by LPS and reduce the expression of inflammatory factor, such as IL-1β, IL-6, and TNF-α, thereby alleviating inflammatory response (Yang et al., 2018). The Phellodendron chinense C.K.Schneid. [Rutaceae] ketone contained in Phellodendron chinense C.K.Schneid. [Rutaceae] can reduce the transcription and translation levels of inflammatory factors, such as NO, IL-6, IL-1β, and MCP-1, and significantly inhibit p38-mediated AP-1 signaling by stabilizing the M-RNA of mitogen-activated protein kinase phosphatase-1 (MKP-1), thereby prolonging the expression time of MKP-1 protein (Gao et al., 2018). We found that only the TCM Bletilla striata (Thunb.) Rchb. f. [Orchidaceae] did not have heat clearing and detoxifying effects. Through searching, we found that *Bletilla striata* (Thunb.) Rchb. f. [Orchidaceae] striata polysaccharide gum in *Bletilla striata* (Thunb.) Rchb. f. [Orchidaceae] is stable in nature and can significantly promote the DNA synthesis of wound surface cells, improve the ability of cell proliferation, and shorten the healing time of the ulcer surface (Lee et al., 2015; Qiu et al., 2007). At the same time, Bletilla striata (Thunb.) Rchb. f. [Orchidaceae] has a good film-forming property, which can benefit drugs with slow-release effects and has a significant effect on the treatment of oral ulcers (Liu and Jiao, 2015). Therefore, a reasonable combination of these Chinese medicines can theoretically enhance anti-inflammatory effects, improve the oral mucosal environment, and ultimately show a significant prevention and treatment effect on CIOM.

This study has certain limitations. First, the QRJD-TCM gargle is limited to the trials of patients receiving chemotherapy. We excluded trials involving radiotherapy or combined chemotherapy to reduce the heterogeneity between the trials, but we recognized that this procedure limited the external effectiveness and applicability of the study results. Therefore, our results may not be applicable to adult patients receiving radiotherapy or combined chemotherapy. Second, although the QRJD-TCM gargle is a decoction, the specific medicine, dosage, preparation method, and use frequency vary. In addition, no separate analysis of differences in interventions between the control group was performed. These limitations may require further study. Third, the characteristics of all patients were heterogeneous, which is a significant limitation of our study. Possible confounding factors included patient's age, chemotherapy regimen, frequency, dosage, type of cancer, and combined interventions. Fourth, most of the included studies did not report adverse events, and verifying the safety of the QRJD-TCM gargle for the prevention and treatment of CIOM was difficult. Fifth, most of the included studies were based on China and rarely involved foreign countries. Thus, determining whether the efficacy of QRJD-TCM gargle for CIOM is applicable to different populations around the world was difficult. Sixth, our current study had not been registered, and thus a small level of bias may have been present. However, we still strictly followed the steps of systematic review in the conduct of the meta-analysis. Despite these limitations, the results may be useful to clinicians.

## Conclusion

The results of this study indicate that QRJD-TCM gargle is more effective than CWMM in the prevention and treatment of CIOM. QRJD-TCM gargle can be used as an alternative or complementary therapy for CWMM in the whole course of chemotherapy. However, owing to the small sample size and poor methodological quality of the included studies, further standard, double-blind, and multicenter randomized controlled studies are needed to confirm the clinical value of QRJD-TCM gargle in the treatment of CIOM. In addition, future studies should focus on how to properly combine Chinese medicines for the preparation of QRJD-TCM gargle that can exert good therapeutic effect against CIOM.

## Data Availability

The original contributions presented in the study are included in the article/Supplementary Material, further inquiries can be directed to the corresponding author.

## Abbreviations

CIOM, Chemotherapy-Induced Oral Mucositis; TCM, traditional Chinese medicine; QRJD-TCM, Heat-Clearing (Qingre) and Detoxifying (Jiedu) Traditional Chinese Medicine; CNKI, China National Knowledge Infrastructure; VIP, Chinese Scientific Journal Database; RCTs, randomized controlled trials; CWMM, conventional western medicine mouthwashes; OM, oral mucositis; PRISMA, Systematic Review and Meta-Analysis; OR, odds ratio; CI, confidence interval; I2, I-square.
