# Supplementary material for: The Efficacy of Heat-Clearing (Qingre) and Detoxifying (Jiedu) Traditional Chinese Medicine Gargle for Chemotherapy-Induced Oral Mucositis: A Systematic Review and Meta-Analysis
Source: Front Pharmacol. 2021 Mar 29;12:627628. doi: 10.3389/fphar.2021.627628 (PMC8040107; doi:10.3389/fphar.2021.627628)
Supplement: Supplementary file 1 [file datasheet1.docx]

**Supplement 1 shows the search strategy used for the systematic review**

Search of Cochrane Library

Date Run: 20/10/2020 16:19:36

Comment:

ID Search Hits

#1 (oral mucositis):ti,ab,kw OR (oral ulcer):ti,ab,kw (Word variations have been searched) 8175

#2 (chemotherapy):ti,ab,kw (Word variations have been searched) 76020

#3 ("traditional Chinese medicine") OR ("Chinese herbal medicine") OR (herbs) OR (Heat-Clearing) OR (Detoxifying) 15885

#4 (Qingre) OR (Jiedu) 287

#5 #3 OR #4 16021

#6 #1 AND #2 AND #5 8

Search strategy for Wangfang

题名: "化疗" and (题名: "口腔溃疡" or "口腔黏膜炎") and (主题: "中药" or "中草药" or "草药" or "清热" or "解毒") . 69 literatures were searched.
